# Optimizing cold storage of *Sitotroga cerealella* eggs for the mass rearing of *Trichogrammatoidea bactrae*: Effects on reproductive performance of parent and progeny

**DOI:** 10.1038/s41598-026-64919-5

**Published:** 2026-08-08

**Authors:** Hend O. Mohamed, Walaa E. Gamil, Hassan F. Dahi

**Affiliations:** 1 Biological Control Research Department, Plant Protection Research Institute, Agricultural Research Center, Giza, Egypt; 2 Cotton Leafworm Research Department, Plant Protection Research Institute, Agricultural Research Center, Giza, Egypt

**Keywords:** Biological quality, Host egg, Mass production, Storage duration, Trichogrammatidae, Ecology, Ecology, Zoology

## Abstract

Improving effective cold storage techniques for maintaining factitious host eggs without adversely affecting their quality and the performance of emerged parasitoids is essential. Therefore, this study investigated the effects of different storage temperatures (–2 °C, 2 °C, 4 °C, and 8 °C) and durations (1–25 days) on the quality of *Sitotroga cerealella* Olivier (Lepidoptera: Gelechiidae) eggs and the reproductive performance of both parents and offspring of *Trichogrammatoidea bactrae* Nagaraja (Hymenoptera: Trichogrammatidae). This parasitoid demonstrated the ability to parasitize cold-stored eggs, albeit at varying rates influenced by temperature and storage duration. Storage at 4 °C sustained egg quality and parasitoid performance for up to 25 days, however, optimal results of high parasitism rates (> 93%), adult emergence (> 96%), and female proportions (> 75%) were specifically maintained for 19 days. Additionally, storage at 2 °C held host suitability for 13 days. These trends were consistent for both parental and progeny generations. In contrast, eggs stored at 8 °C, exhibited parasitism rates of approximately 85% for 9 days, dropping to 54% by day 17 and failing completely by day 19. Conversely, storage at – 2 °C caused rapid deterioration of host egg quality after 5 days, reducing parasitism (83.47%) and significantly reducing parental (F_0_) performance. However, parasitoids that successfully emerged did not exhibit detectable carry-over effects in the subsequent generation (F_1_). Notably, while parasitism rates declined at – 2 °C and 8 °C with extended storage, adult emergence remained consistently high (approximately 92–98%) across all durations. Under these storage conditions, parasitoid emergence was predominately female-biased (65–80%) in both generations. However, at – 2 °C after 9 days, the ratio shifted sharply to male-biased (52%) in F_0_ generation only, indicating an adverse effect on sex allocation, with no transgenerational effect in the F_1_ progeny. These findings identify 4 °C for 19 days as the optimal protocol for sustaining *S. cerealella* egg quality and *T. bactrae* efficacy, followed by 2 °C for 13 days. Selecting appropriate storage conditions is valuable for effective augmentative biological control programs.

## Introduction

Worldwide, trichogrammatids are among the most widely used and extensively studied egg parasitoids as vital biological control agents against a diverse range of insect pests threatening economically important crops such as cotton, maize, sugarcane, tomato, and potato^1^. Their economic importance is attributed to their polyphagous nature, ease of mass rearing, and widespread geographic distribution. Among these, *Trichogrammatoidea bactrae* Nagaraja (Hymenoptera: Trichogrammatidae) is considered one of the most promising species for Integrated Pest Management (IPM) programs. It was introduced into California from Queensland, Australia, in 1985 as a potential biological control agent against the pink bollworm (PBW), *Pectinophora gossypiella* (Saunders)^2^. The genus *Trichogrammatoidea* comprises approximately 25 species distributed primarily throughout the Oriental, African, and Australian regions. Due to its high ecological plasticity, *T. bactrae* has been successfully introduced and established in various other biogeographic regions^2^. In Egypt, this parasitoid species has demonstrated remarkable adaptability to the local climate, contributing significantly to the suppression of cotton bollworms in Upper Egypt^3^. It is highly effective against lepidopteran pests, particularly those infesting field crops and stored commodities^3–7^. Under standard rearing conditions, *T. bactrae* exhibits high thermal tolerance (up to 35˚C), a short life cycle, high parasitism and adult emergence rates, and a female-biased sex ratio^5,6,8^. Furthermore, early suppression of pest populations at the egg stage reduces reliance on chemical insecticides and supports the environmental sustainability of pest management^8^. Consequently, the mass production of *T. bactrae* has become a global standard practice to provide high-quality individuals for large-scale augmentative field releases. However, the high costs associated with rearing parasitoids on natural egg hosts or other target pests such as cotton bollworms or fall armyworm remain a major challenge, maintaining continuous host colonies requires substantial labor, space, artificial diet, and environmental control^9–11^. To combat this, eggs of the Angoumois grain moth, *Sitotroga cerealella* Olivier (Lepidoptera: Gelechiidae), are commonly used as a factitious host under laboratory conditions^1^, due to their efficient rearing on cereal grains, high fecundity, and high suitability for parasitoid development. Maintaining a continuous and stable supply of healthy host eggs is vital for the successful mass production of trichogrammatids^12^. Thus, improving storage techniques for fresh eggs has gained significant research attention to facilitate colony management and maintain continuous parasitoid production for field releases^13^. Because the host egg is the sole nutrient source for the developing immature parasitoid, its quality is a critical determinant of parasitoid fitness^14^. Any deterioration in the nutritional content during storage can negatively affect the parasitoid’s development and reproductive performance^13^. Specifically, prolonged storage of eggs can sharply decline their quality and fitness, detrimentally affecting host recognition, acceptance, parasitism, and subsequent parasitoid emergence rates^15–18^. While, many studies have investigated the cold storage of parasitized eggs of various *Trichogramma* species^19–21^, research addressing the impact of storage fresh (un-parasitized) eggs on the biological fitness and progeny performance of *T. bactrae* remains comparatively limited. Therefore, the present study evaluated the impact of different cold storage temperatures from sub-zero (−2 °C) to sub-optimal (8 °C), and different exposure periods on the reproductive performance of *T. bactrae*. Sub-zero temperatures were examined to determine the lower thermal limits for stockpiling fresh host eggs while maintaining their suitability for parasitoid development. Additionally, the effectiveness of these storage conditions on the next generation (F_1_ progeny) in terms of parasitism rate, adult emergence rate, and female ratio was assessed for optimizing the mass rearing protocols under laboratory conditions. Such detailed knowledge is crucial for improving the flexibility of mass-rearing programs by allowing temporary storage of host eggs to facilitate the availability of high-quality parasitoids during periods of increased pest incidence and field release demand.

## Results

### Effect of cold stored ***S. cerealella*** eggs on reproductive performance of ***T. bactrae*** (F_0_ generation)

#### Parasitism rate

The parasitoid, *T. bactrae* successfully parasitized *S. cerealella* eggs under storage conditions, with parasitism rates significantly influenced by storage temperature (*F*_3,447_ = 154.21; *p* < 0.001), storage duration (*F*_12,447_ = 56.57; *p* < 0.001), and their interaction (*F*_36,447_ = 9.31, *p* < 0.001) (Table 1). The mean parasitism rate on fresh eggs was 94.93% compared to 96.38% for eggs stored at −2 °C for 3 days only, revealing that short-term sub-zero storage did not adversely affect host egg suitability. However, prolonged storage at −2 °C progressively reduced parasitism rates, declining to 51.98% by day 15 (Table 2). Beyond this period, no blackened (parasitized) eggs were noticed, indicating severe deterioration in egg suitability and quality under extended storage. In contrast, eggs stored at 2 °C and 4 °C provided superior performance, demonstrating that these temperatures were optimal for maintaining *T. bactrae* parasitism across longer storage durations. Parasitism rates remained consistently high (> 91%) for up to 13 days at 2 °C and > 93% for up to 19 days at 4 °C, before declining to approximately 80% after 21 days and 50% after 25 days. Conversely, eggs stored at 8 °C displayed an earlier decline in host suitability than those stored at 2 °C and 4 °C, with parasitism rates decreasing significantly from approximately 91% during one day of storage to around 54% by day 17.

**Table 1:** General Linear Model (GLM) analysis of the effects of storage temperature, duration, and their interaction on the parasitism rates of *Trichogrammatoidea bactrae*.

Source of Variation	Sum of Squares (SS)	Degree of freedom (df)	F		*P*-value	
Temperature (T)	845.20	3	154.21		< 0.001*	
Duration (D)	1240.15	12	56.57		< 0.001*	
Interaction (T × D)	612.40	36	9.31		< 0.001*	
Error (Within)	723.45	396				
Total	3421.20	447				

**Table 2:** Parasitism rates of *Trichogrammatoidea bactrae* on cold-stored *Sitotroga cerealella* eggs at different temperatures and storage periods.

Parasitism rates (%)				
Storage periods (days)	Storage temperatures			
	−2 ^o^ C	2 ^o^ C	4 ^o^ C	8 ^o^ C
Control	94.93 ± 0.65^a^	94.93 ± 0.65^ab^	94.93 ± 0.65^bcd^	94.93 ± 0.65^a^
1	96.33 ± 0.33^aA^	96.93 ± 0.5^aA^	95.81 ± 0.15^cA^	91.44 ± 0.79^bB^
3	96.38 ± 0.37^aB^	97.88 ± 0.16^aA^	96.36 ± 0.13^bB^	88.11 ± 0.96^cC^
5	83.47 ± 1.64^bD^	94.91 ± 0.35^bB^	98.25 ± 0.07^aA^	87.90 ± 0.66^cC^
7	71.12 ± 2.08^cD^	92.84 ± 0.42c^dB^	96.42 ± 0.17^bA^	86.46 ± 0.72^cdC^
9	67.03 ± 0.99^cD^	93.88 ± 0.25^cB^	95.66 ± 0.28^cdA^	84.77 ± 0.61^dC^
11	56.97 ± 1.15^dD^	92.05 ± 0.42^dB^	94.88 ± 0.36^dA^	75.56 ± 1.59^eC^
13	58.08 ± 0.91^dD^	91.97 ± 0.59^dB^	93.57 ± 0.21^eA^	71.35 ± 0.73^fC^
15	51.98 ± 1.53^eD^	66.89 ± 1.12^eB^	96.95 ± 0.29^bA^	60.15 ± 1.22^gC^
17	0.00 ± 0.00^fD^	67.50 ± 1.55^eB^	93.41 ± 0.47^eA^	53.94 ± 0.79^hC^
19	0.00 ± 0.00^fC^	39.70 ± 0.82^fB^	93.01 ± 0.36^eA^	0.00 ± 0.00^iC^
21	0.00 ± 0.00^f^	0.00 ± 0.00^g^	80.42 ± 1.03^f^	0.00 ± 0.00^i^
25	0.00 ± 0.00^f^	0.00 ± 0.00^g^	49.97 ± 2.65^g^	0.00 ± 0.00^i^
F	**220.12**	**643.08**	**265.47**	**222.59**
*P*	**< 0.0001**	**< 0.0001**	**< 0.0001**	**< 0.0001**

Means ± SE sharing the same lowercase letters in the same columns and same uppercase letters within the rows are statistically insignificant (Tukey HSD test, *p* > 0.05), (*n* = 10 replicates).

#### Developmental period

The emergence time required for the development of *T. bactrae* remained relatively consistent across all storage treatments, with mean values ranging from 9.6 to 11.3 days compared to control group (Table 3). The interaction between storage temperature and duration was non-significant (*F*_36,447_ = 0.88; *p* = 0.6657) on the developmental timing of the parasitoid, revealing that once parasitism was successful, host storage conditions did not significantly accelerate or delay internal development.

**Table 3:** Developmental periods of *Trichogrammatoidea bactrae* from cold-stored *Sitotroga cerealella* eggs at different temperatures and storage periods.

Developmental periods (days)				
Storage periods (days)	Storage temperatures			
	−2 ^o^ C	2 ^o^ C	4 ^o^ C	8 ^o^ C
Control	**10.2 ± 0.25** ^ab^			
1	10.3 ± 0.3^b^	10.2 ± 0.25^ab^	10 ± 0.26^ab^	10.2 ± 0.25^a^
3	10.6 ± 0.22^b^	10.5 ± 0.17^a^	10.3 ± 0.37^ab^	10.7 ± 0.21^a^
5	10.8 ± 0.25^ab^	10.7 ± 0.21^a^	10.3 ± 0.37^ab^	10.3 ± 0.21^a^
7	10.5 ± 0.17^b^	10.4 ± 0.27^a^	10.5 ± 0.40^ab^	10.5 ± 0.17^a^
9	11.3 ± 0.15^a^	10.6 ± 0.27^a^	10.3 ± 0.37^ab^	10 ± 0.33^a^
11	10.3 ± 0.26^b^	10.6 ± 0.22^a^	10.1 ± 0.28^ab^	10.3 ± 0.26^a^
13	10.5 ± 0.31^b^	10.5 ± 0.27^a^	10.1 ± 0.28^ab^	9.9 ± 0.38^a^
15	10.4 ± 0.30^b^	9.6 ± 0.22^b^	10.2 ± 0.25^ab^	10 ± 0.33^a^
17	0.00 ± 0.00^c^	10.4 ± 0.30^a^	10.5 ± 0.17^a^	10 ± 0.26^a^
19	0.00 ± 0.00^c^	10.6 ± 0.22^a^	10.5 ± 0.17^a^	0.00 ± 0.00^c^
21	0.00 ± 0.00^c^	0.00 ± 0.00^c^	10.1 ± 0.35^ab^	0.00 ± 0.00^c^
25	0.00 ± 0.00^c^	0.00 ± 0.00^c^	9.8 ± 0.13^b^	0.00 ± 0.00^c^
*F*	**1.7807**	**1.6007**	**0.5135**	**1.0395**
*P*	**0.0928**	**0.1174**	**0.9024**	**0.4144**

Means ± SE sharing the same lowercase letters in the same columns are statistically insignificant (Tukey HSD test, *p* > 0.05), *n* = 10 replicates. There is no significant difference between any means within the same rows.

#### Adult emergence rate

High emergence rates of *T. bactrae* wasps (approximately 92–98%) were observed across all storage treatments, compared to the control (97%) (Table 4). Emergence rates were significantly influenced by storage temperature (*F*_3,447_ = 12.64; *p* < 0.0001), duration (*F*_12,447_ = 24.42; *p* < 0.0001), and their interaction (*F*_36,447_ = 2.93; *p* < 0.0001) (Table 5). Notably, the highest rates (exceeding 95%) were recorded at 4 °C for the majority of the storage duration, with only a gradual decrease to 85.63% at 25 day. This was followed by the 2 °C, which maintained high viability for 17 days. Both 2 °C and 4 °C exhibited their suitability for long-term egg storage without detrimental effects on *T. bactrae* emergence. Although, parasitism rates declined sharply at −2 °C and 8 °C over time, the emergence rates remained high (above 92%) for all storage durations (Table 2).

**Table 4:** Adult emergence rates of *Trichogrammatoidea bactrae* from cold-stored *Sitotroga cerealella* eggs under different temperatures and periods.

Adult emergence rates (%)						
Storage periods (days)	**Storage temperatures**					
	**−2** ^o^ **C**	**2** ^o^ **C**		**4** ^o^ **C**		**8** ^o^ **C**
Control	**97.05 ± 0.26** ^bc^					
1	97.26 ± 0.19^bA^	97.87 ± 0.30^bA^		96.95 ± 0.24^bA^		95.30 ± 0.34^cB^
3	96.36 ± 0.46^bcB^	93.00 ± 0.67^dC^		98.32 ± 0.19^aA^		97.34 ± 0.28^bcB^
5	97.96 ± 0.22^aB^	98.66 ± 0.20^aA^		97.07 ± 0.18^bB^		93.74 ± 0.23^dC^
7	95.87 ± 0.65^bcA^	96.90 ± 0.33^cA^		95.92 ± 0.33^cA^		96.20 ± 0.48^cA^
9	96.43 ± 0.48^bcB^	93.25 ± 0.42^dC^		95.63 ± 0.27^cB^		98.34 ± 0.12^aA^
11	96.42 ± 0.48^bcB^	97.14 ± 0.53^bcA^		98.06 ± 0.16^aA^		96.14 ± 0.35^cB^
13	95.15 ± 0.35^cC^	97.65 ± 0.23^bcA^		96.20 ± 0.22^cB^		97.74 ± 0.15^bA^
15	92.28 ± 0.56^dB^	92.86 ± 0.78^dB^		94.59 ± 0.22^dA^		95.89 ± 0.65^cA^
17	0.00 ± 0.00^e^	97.16 ± 0.33^bcA^		95.69 ± 0.38^cB^		92.54 ± 0.23^eC^
19	0.00 ± 0.00^e^	87.29 ± 0.87^eB^		96.89 ± 0.52^bcA^		0.00 ± 0.00^f^
21	0.00 ± 0.00^e^	0.00 ± 0.00^f^		91.91 ± 0.80^e^		0.00 ± 0.00^f^
25	0.00 ± 0.00^e^	0.00 ± 0.00^f^		85.63 ± 1.19^f^		0.00 ± 0.00^f^
*F*	**14.1599**	**46.2639**		**49.1412**		**26.9237**
*P*	**< 0.0001**	**< 0.0001**		**< 0.0001**		**< 0.0001**

Means ± SE sharing the same lowercase letters in the same columns and same uppercase letters within the rows are statistically insignificant (Tukey HSD test, *p* > 0.05), *n* = 10 replicates.

**Table 5:** General Linear Model (GLM) analysis of the effects of storage temperature, duration, and their interaction on adult emergence rates of *Trichogrammatoidea bactrae*.

Source of Variation	Degree of freedom (df)	Sum of Squares (SS)	F	*P*-value
Temperature (T)	3	148.22	12.64	**< 0.0001**
Duration (D)	12	1145.60	24.42	**< 0.0001**
Interaction (T × D)	36	412.35	2.93	**< 0.0001**
Error	396	1548.12		
Total	447	3254.29		

#### Female proportion

The proportion of *T. bactrae* females was significantly influenced by host egg storage conditions (*p* < 0.0001) (Table 6). Overall, parasitoid emergence exhibited a female-biased of approximately 65% to 80% under various storage conditions compared to the control group (78.75%). However, eggs stored at −2 °C for 9 days, the female ratio sharply declined to an average of 48%, shifting toward a male-bias and indicating an adverse effect on sex allocation (Table 7). The highest and most stable female ratios were consistently observed at 4 °C, where the proportions remained above 75% even after prolonged durations. Moderate fluctuations in female ratio (63 to 73%) were noted at 2 °C, reaching its minimum (57.08%) after 19 days. At 8 °C, slightly variable female proportions were observed over extended periods, ranging from approximately 65% to 74%.

**Table 6:** General Linear Model (GLM) analysis of the effects of storage temperature, duration, and their interaction on the proportion of *Trichogrammatoidea bactrae* females.

Source of Variation	Degree of freedom (df)	Sum of Squares (SS)	F	*P*-value
Temperature (T)	3	2984.1	47.16	**< 0.0001**
Duration (D)	12	1856.4	7.33	**< 0.0001**
Interaction (T × D)	36	2145.8	2.83	**< 0.0001**
Error	396	11051.1		
Total	447	18037.4		

**Table 7:** Percentages of *Trichogrammatoidea bactrae* females emerged from cold-stored *Sitotroga cerealella* eggs at different temperatures and periods.

Female ratio (%)						
Storage periods (days)	**Storage temperatures**					
	**−2** ^o^ **C**	**2** ^o^ **C**		**4** ^o^ **C**		**8** ^o^ **C**
Control	**78.75 ± 1.45** ^**ab**^					
1	72.75 ± 0.48^cA^	66.00 ± 0.25^eB^		75.00 ± 0.46^bA^		73.67 ± 1.55^bA^
3	71.17 ± 1.07^cA^	63.25 ± 1.74^eB^		74.58 ±.02^bA^		72.33 ± 0.98^bcA^
5	73.33 ± 0.93^bcB^	71.25 ± 0.22^cdB^		77.67 ± 1.14^aA^		72.17 ± 1.25^cB^
7	68.33 ± 0.69^dC^	72.75 ± 1.46^bcB^		77.50 ± 1.16^bA^		68.50 ± 1.48^dBC^
9	48.33 ± 2.33^eC^	73.33 ± 1.61^bcB^		79.42 ± 1.08^aA^		72.17 ± 1.13^cB^
11	74.83 ± 0.80^bA^	72.17 ± 0.60^bcB^		76.50 ± 0.87^bA^		66.08 ± 1.05^dC^
13	75.58 ± 1.72^abcAB^	73.67 ± 0.62^bB^		79.42 ± 1.01^aA^		64.92 ± 0.54^dC^
15	72.00 ± 0.86^cB^	71.00 ± 1.29^cdB^		75.67 ± 0.99^bA^		70.17 ± 0.94^cdB^
17	0.00 ± 0.00^d^	68.08 ± 1.54^deB^		79.92 ± 1.13^aA^		67.17 ± 1.17^dB^
19	0.00 ± 0.00^d^	57.08 ± 1.95^fB^		75.33 ± 1.77^bA^		0.00 ± 0.00^e^
21	0.00 ± 0.00^d^	0.00 ± 0.00^g^		77.83 ± 1.02^a^		0.00 ± 0.00^e^
25	0.00 ± 0.00^d^	0.00 ± 0.00^g^		80.50 ± 0.88^a^		0.00 ± 0.00^e^
*F*	**48.0309**	**20.5714**		**3.1998**		**11.9666**
*P*	**< 0.0001**	**< 0.0001**		**0.0004**		**< 0.0001**

Means ± SE sharing the same lowercase letters in the same columns and same uppercase letters within the rows are statistically insignificant (Tukey HSD test, *p* > 0.05), *n* = 10 replicates.

### Fitness of the subsequent generation (F_1_ generation)

The biological efficacy of offspring was assessed to detect potential trans-generational effects in terms of parasitism, adult emergence, and female proportions (Tables 8 and 9). Parasitism rates in progeny were significantly influenced by parental storage temperature (*F*_3,447_ = 38.45; *p* < 0.001) and duration (*F*_12,447_ = 6.90; *p* < 0.001). Generally, progeny parasitism remained consistently high (91 to 98%) without notable deterioration in parasitoid fitness for most treatments, except at the high storage temperature of 8 °C (Table 8a). The highest rates in progeny were observed from *T. bactrae* parents reared on eggs stored at 4 °C (95–98%), followed closely by the 2 °C, and − 2 °C (91–97%), with minor fluctuations compared to the control (94.64%). Although parental parasitism at −2 °C gradually declined after 5 days, the F_1_ progeny still exhibited high rates (91–96%). However, a critical performance failure was recorded in offspring from eggs stored at 2 °C for 19 days; all emerged females adults (57%) failed to parasitize fresh eggs, resulting in zero F_1_ offspring. In contrast, offspring from the 8 °C treatment revealed variations in parasitism rates with extended storage, particularly after 7 days, compared to the control group. The interaction effect between storage temperature and duration (*p* < 0.0001) indicated that stockpiling eggs at 4 °C and 2 °C for the long-term maintained high parasitism rates, while sub-zero (−2 °C) or suboptimal (8 °C) storage temperatures caused detrimental effects on parents. Furthermore, offspring emergence followed a positive trend, with high rates (92–98%) recorded across all egg storage conditions. Nevertheless, two-way ANOVA confirmed significant variations of parental temperature (*F*_3,447_ = 84.34; *p* < 0.0001) and duration (*F*_12,447_ = 12.52; *p* < 0.0001) on F_1_ emergence (Table 8b). A return to a characteristic female-biased sex ratio in the progeny was observed, with females relatively higher than those in the parents under extended storage (Table 9). Specifically, the parental female proportion reared on −2℃ eggs for 9 days dropped to 48%, their subsequent offspring successfully returned to a healthy female-biased ratio of 75.6%. Eggs stored at 4 °C exhibited the best performance of the parasitoid, demonstrating consistently high female rates (73–79%) even over prolonged durations. Cold storage conditions significantly influenced (*F*_36,447_ *=*1.83; *p* = 0.0028) the female proportions in the progeny.

**Table 8:** Parasitism and adult emergence rates of *Trichogrammatoidea bactrae* in the F_1_ progeny from parents reared on cold-stored *Sitotroga cerealella* eggs under different temperatures and storage periods.

Storage period (days)	a- Parasitism rates (%)							b- Adult emergence rates (%)						
	−2^o^C	2^o^C		4^o^C		8^o^C		−2^o^C		2^o^C		4^o^C		8^o^C
Control	**94.64 ± 0.42** ^**cd**^							**96.14 ± 0.43** ^**abc**^						
1	94.55 ± 0.37^cdC^	94.77 ± 0.42^dC^		98.27 ± 0.11^abA^		97.25 ± 0.13^aB^		96.81 ± 0.24^bB^		95.47 ± 0.22^cC^		97.91 ± 0.22^bcA^		95.69 ± 0.30^cC^
3	96.05 ± 0.47^abA^	91.13 ± 0.75^eC^		96.71 ± 0.20^dA^		94.44 ± 0.48^cdB^		97.20 ± 0.21^bB^		94.44 ± 0.24^dD^		95.92 ± 0.38^dC^		98.32 ± 0.14^aA^
5	92.73 ± 0.45^efC^	98.06 ± 0.22^aA^		96.47 ± 0.24^dB^		95.69 ± 0.40^bcB^		98.50 ± 0.28^aA^		95.54 ± 0.45^bcdB^		95.79 ± 0.37^dB^		95.28 ± 0.38^cdB^
7	91.03 ± 0.51^fC^	96.48 ± 0.26^cB^		98.61 ± 0.10^aA^		88.39 ± 0.85^fD^		95.80 ± 0.11^cC^		97.26 ± 0.27^aB^		98.39 ± 0.14^aA^		94.12 ± 0.56^dD^
9	93.00 ± 0.76^deB^	94.20 ± 0.38^dB^		98.24 ± 0.16^abA^		89.33 ± 0.60^efC^		97.28 ± 0.41^bAB^		97.39 ± 0.10^aB^		98.15 ± 0.12^abA^		94.84 ± 0.40^cdC^
11	96.80 ± 0.25^aA^	97.44 ± 0.18^bA^		96.79 ± 0.38^cdA^		91.26 ± 0.42^eB^		95.44 ± 0.37^cBC^		96.27 ± 0.25^bB^		95.43 ± 0.18^dC^		97.62 ± 0.29^abA^
13	94.99 ± 0.60^bcB^	96.00 ± 0.11^cB^		97.18 ± 0.16^cA^		78.27 ± 1.08^hC^		92.26 ± 0.44^eC^		96.98 ± 0.31^abA^		95.42 ± 0.11^dB^		95.78 ± 0.50^cAB^
15	91.04 ± 0.45^fC^	92.53 ± 0.51^eB^		95.27 ± 0.32^dA^		84.23 ± 1.80^gD^		86.37 ± 0.82^fB^		97.66 ± 0.21^aA^		97.43 ± 0.21^cA^		96.98 ± 0.24^bA^
17	0.00 ± 0.00^g^	91.99 ± 0.49^eB^		97.26 ± 0.14^cA^		88.48 ± 1.15^fC^		0.00 ± 0.00^g^		95.36 ± 0.63^bcdB^		98.60 ± 0.08^aA^		98.23 ± 0.48^aA^
19	0.00 ± 0.00^g^	0.00 ± 0.00^f^		97.48 ± 0.34^bc^		0.00 ± 0.00^i^		0.00 ± 0.00^g^		0.00 ± 0.00^e^		97.27 ± 0.30^c^		0.00 ± 0.00^e^
21	0.00 ± 0.00^g^	0.00 ± 0.00^f^		96.54 ± 0.18^d^		0.00 ± 0.00^i^		0.00 ± 0.00^g^		0.00 ± 0.00^e^		97.52 ± 0.23^c^		0.00 ± 0.00^e^
25	0.00 ± 0.00^g^	0.00 ± 0.00^f^		97.05 ± 0.14^cd^		0.00 ± 0.00^i^		0.00 ± 0.00^g^		0.00 ± 0.00^e^		95.51 ± 0.30^d^		0.00 ± 0.00^e^
*F*	**17.2489**	**33.5800**		**21.0827**		**44.4627**		**9.3970**		**21.2093**		**21.2093**		**13.3652**
*P*	**< 0.0001**	**< 0.0001**		**< 0.0001**		**< 0.0001**		**< 0.0001**		**< 0.0001**		**< 0.0001**		**< 0.0001**

Means ± SE sharing the same lowercase letters in the same columns and same uppercase letters within the rows are statistically insignificant (Tukey HSD test, *p* > 0.05), (*n* = 10 replicates).

**Table 9:** Percentages of *Trichogrammatoidea bactrae* females in the F_1_ progeny emerging from parents reared on cold-stored *Sitotroga cerealella* eggs under different temperatures and storage periods.

Female ratio (%)							
Storage periods (days)	**Storage temperatures**						
	**−2** ^**o**^ **C**	**2** ^o^ **C**		**4** ^o^ **C**			**8** ^o^ **C**
Control	**80.90 ± 1.99** ^**a**^						
1	73.10 ± 1.29^deA^	65.20 ± 1.73^dB^		73.80 ± 2.60^abA^			74.40 ± 1.83^aA^
3	73.10 ± 0.67^deA^	72.60 ± 0.69^cA^		73.90 ± 1.24^bA^			72.30 ± 0.82^bA^
5	74.80 ± 0.90^cdA^	72.60 ± 0.37^cA^		75.60 ± 1.84^abA^			72.30 ± 1.09^bA^
7	75.60 ± 0.50^bcA^	75.00 ± 1.25^abcA^		77.40 ± 1.46^abA^			74.90 ± 0.69^aA^
9	79.00 ± 0.79^aA^	74.30 ± 0.60^bA^		77.20 ± 2.15^abA^			68.40 ± 1.43^cB^
11	75.60 ± 0.54^bcA^	75.70 ± 1.34^abcA^		76.70 ± 1.48^abA^			70.50 ± 0.62^bcB^
13	70.50 ± 2.08^deA^	71.90 ± 1.46^cA^		75.00 ± 1.81^abA^			73.00 ± 1.53^bA^
15	71.50 ± 0.83^eB^	71.90 ± 1.39^cB^		79.30 ± 1.27^aA^			67.60 ± 1.47^cC^
17	0.00 ± 0.00^f^	75.40 ± 0.69^aA^		76.20 ± 1.91^abA^			70.90 ± 0.48^bcB^
19	0.00 ± 0.00^f^	0.00 ± 0.00^e^		77.80 ± 0.66^a^			0.00 ± 0.00^d^
21	0.00 ± 0.00^f^	0.00 ± 0.00^e^		74.70 ± 1.48^ab^			0.00 ± 0.00^d^
25	0.00 ± 0.00^f^	0.00 ± 0.00^e^		74.90 ± 2.34^ab^			0.00 ± 0.00^d^
*F*	**6.5485**	**8.5587**		**1.3135**			**7.3077**
*P*	**< 0.0001**	**< 0.0001**		**0.2199**			**< 0.0001**

Means ± SE sharing the same lowercase letters in the same columns and same uppercase letters within the rows are statistically insignificant (Tukey HSD test, *p* > 0.05), *n* = 10 replicates.

## Discussion

Optimizing cold storage techniques for host eggs without adverse effects on their quality has become a focal point in biological control research^13,18^. This technique is essential for improving operational flexibility of mass-rearing programs and reducing production costs by extending egg shelf life, preserving sufficient parasitoid numbers when needed for field releases, and minimizing the waste of biological materials^22^. Improper storage can significantly diminish host suitability, recognition, and acceptance, contributing to the deficiency of parasitoid performance. Previous studies have demonstrated that prolonged storage can negatively affects parasitoid efficacy driven by storage temperature, duration, egg host, and *Trichogramma* species^12,23,24^. Consequently, the present study aimed to identify the most suitable cold storage protocol for *S. cerealella* eggs that maintains the biological performance of *T. bactrae* and its progeny. Parasitism rate serve as a primary indicator of parasitoid performance, representing the female’s capability to detect host chemical cues and tolerate physical compositions changes in the egg, such as shifts in color, size, and shape^25^. Our results demonstrated that 4 °C was the most suitable storage temperature, maintaining *S. cerealella* egg quality for up to 25 days and ensuring high parasitism rates (> 93%) for 19 days, followed by 2 °C for 13 days. At 4 °C, metabolic activity may be sufficiently suppressed to reduce the depletion of energy and delay senescence, thus sustaining host quality during extended storage periods^26,27^. Conversely, the sharp decline in parasitism when eggs stored at 8 °C after 17 days (dropping to 0% at 19 days) suggests a “chemical tipping point” where the host egg becomes unrecognizable^18^. These findings relatively align with previous reports by^13,27,28^ who noted that storage of the factitious eggs, *Corcyra cephalonica* (Stainton) at 4 °C should not exceed 15 days, because long-term storage led to yolk deterioration. Such deterioration reduced the nutritional value available to the *Trichogramma* embryos^29^ and may be related to changes in internal nutrient compositions such as alanine, glutamine, glucose, and acetate alongside increases in total concentrations of amino acids, carbohydrates, nucleic acid, and organic acid^13^. Data for *S. cerealella* eggs stored at (−2 °C) > 5 days reached a critical turning point in egg quality deterioration, subsequently affecting *T. bactrae* fitness. Sub-zero storage temperatures may induce physiological alterations in host eggs, including water content loss, increased free amino acids and osmotic pressure, and soluble solid nutrients stickiness within the host eggs as reported in other host egg species such as *C. cephalonica* under low storage conditions^27^. These changes may hinder nutrient absorption during the parasitoid embryonic development. On contrary, Özder^30^ noted that *Ephestia kuehniella* (Zeller) factitious eggs could be stored at 0 °C for 14 days with high parasitism (91%) by *Trichogramma cacoeciae*, followed by 4 °C for 7 days only (92%), and 8 °C was the least suitable storage temperatures. Supportive to this finding, Siam et al.^23^ observed no negative effects on *Trichogramma evanescens* fitness when *S. cerealella* eggs stored at 5 °C for 5 and 10 days, but all parasitoid biological quality dropped after prolonged durations for 30 days.

Parasitoid emergence and female proportion are also vital biological aspects for successful large-scale production system. In the present study, *T. bactrae* maintained high emergence rates (92–98%) and female-biased sex ratios in both parental and offspring generations under storage conditions. In this concept, Zhang et al.^24^ noted that *Trichogramma chilonis* emergence rate remained < 90% when stored *C. cephalonica* eggs at 4 °C for up to 20 days, but dropped to ≤ 80% after 25 days. Similarly, Siam et al.^23^ recorded a gradual reduction in *T. evanescens* emergence from refrigerated *S. cerealella* eggs stored at 5 °C after prolonged storage (30 days). Adverse effects on adult emergence rate, fecundity, and female’s ratio in progeny were also reported on stored *E. kuehniella* eggs over 30 days^31^. Differences among species-specific responses to cold storage have also been documented on parasitoids reared on refrigerated *E. kuehniella* eggs at 5 °C. de Carvalho Spinola-Filho et al.^29^ exhibited that the suitable storage duration varied considerably among *Trichogrammatoidea annulata* and 9 *Trichogramma* species (*T. acacioi*, *T. atopovirilia*, *T. benneti*, *T. brasiliensis*, *T. bruni*, *T. demoraesi*, *T. galloi*, *T. pretiosum*, and *T. soaresi*), ranging from 13 to 23 days based on the examined parasitoid species. On contrary, Özder^30^ found that eggs stored at 0 °C negatively affected *T. cacoeciae* emergence than other storage temperatures (4 °C and 8 °C). These variations in emergence rates suggest that parasitoid responses to cold storage are species-specific and may differ between *Trichogramma* and *Trichogrammatoidea* due to differences in physiological tolerance and adaptation to host quality changes over extended storage durations. Interestingly, our study observed a high female emergence (74.58–80.50%) even after 25 days of storage at 4 °C, and this concept is aligned partially with previous finding conducted at 4–5 °C^23,27^. It is worth mentioning that, no offspring were produced when parental parasitoid emerged from eggs stored at 2 °C for 19 days. This reproductive failure may be due to female parasitoids avoiding oviposition in stored eggs with altered physiological or chemical profiles, such as alanine and glycerol, which often changes during extended cold durations as reported previously by Rivers et al.^32^, although these mechanisms were not directly examined in the present study. Besides, Özder^30^ hypothesized that female parasitoids may adaptively choose to lay more unfertilized male eggs in low quality stored eggs to minimize the reproductive waste “haplodiploid sex-allocation”.

In addition to intrinsic biological parameters such as parasitism, emergence, and sex ratio, behavioral competence is increasingly recognized as a critical determinant of parasitoid field efficacy. Continuous rearing of *T. bactrae* on the factitious host *S. cerealella* may influence behavioral plasticity, olfactory learning, and responsiveness to host-associated semiochemicals. Moreover, the potential effects of extended cold storage on these behavioral traits remain to be elucidated. Therefore, future studies should integrate behavioral assays and functional response analyses using both laboratory and field-collected host eggs to better understand the relationship between laboratory rearing protocols and reliable predictors of field efficacy in augmentative biological control programs^33^.

Ultimately, the effectiveness of cold-storage techniques is highly changed upon low-temperature, storage duration, host egg species, and parasitoid species used. Understanding these interactions is crucial for improving mass-rearing efficiency and supporting successful conservation and augmentative biological control programs against the target pest during crop-growing seasons.

## Conclusion

This study demonstrated that cold storage conditions (temperature and duration) play a key role in maintaining the quality of *S. cerealella* eggs to ensure high parasitism, adult emergence, and female progeny in *T. bactrae*. A moderate storage temperature (4 °C) proved to be the optimal threshold for maintaining host egg vigor and quality, and thus parasitoid reproductive performance over the long-term (up to 25 days), in both parental and offspring generations. Notably, parasitism remained (> 93%) for 19 days, followed by 2 °C for 13 days (> 91%).

## Methods

### Insect and parasitoid rearing

Fresh eggs of *S. cerealella* were obtained from a long-term stock colony reared on wheat grains (*Triticum aestivum* L., var. Seds12) at the *Trichogramma* Mass Rearing Unit, Plant Protection Research Institute (PPRI), Agricultural Research Center (ARC), Assiut Governorate, Upper Egypt. The mass-rearing setup for *S. cerealella* followed the methodology conducted by Hamed and Nadeem (2010)^34^. Eggs were collected daily to serve as factitious hosts for the parasitoid.

The parasitic wasp, *T. bactrae* used in this study was sourced from established laboratory cultures kept at the same rearing unit. To produce the parasitized egg cards " Tricho-cards”, fresh *S. cerealella* eggs (< 24 h) were adhered to self-sticky white A_4_ paper cards and exposed to approximately 500 newly emerged *T. bactrae* adults in cylindrical plastic jars (2 L capacity; 15 cm height × 12 cm diameter) for 24 h to prevent super-parasitism^6,8^. Meanwhile, 10% sugar solution was provided as a nutritional source for the parasitoids. The parasitized egg cards were collected and transferred to clean jars for subsequent experiments. All colonies and experiments were maintained under standard laboratory rearing conditions (25 ± 1 °C, 50–60% relative humidity, and a 14 L: 10D photoperiod)^8^.

### Experimental setup for cold-stored ***S. cerealella*** eggs

Eggs of *S. cerealella* were subjected to a gradient of four cold storage temperatures (−2 °C, 2 °C, 4 °C, and 8 °C) in a refrigerator/incubator to evaluate their effect on host suitability and parasitoid performance. These low temperatures represented a range from sub-zero for testing the impact of severe cold shock and potential freezing injury on host egg safety to sub-threshold temperatures. For each temperature, 12 storage durations were tested (1, 3, 5, 7, 9, 11, 13, 15, 17, 19, 21, and 25 days) to assess the effect of storage eggs on the reproductive quality of *T. bactrae*, following the methodology conducted by Siam et al. (2019)^23^. Following each storage interval, host egg cards (1 × 1 cm, containing approximately 0.01 g of eggs) were introduced to *T. bactrae* adults at a ratio of 3:1 (3 stored host cards to 1 parasitized card) within 500 mL transparent plastic jars covered with fine mesh ventilation, under laboratory conditions. Non-stored (Fresh) eggs served as the control group and were maintained under the same laboratory conditions for comparison with the cold-storage treatments. To maximize parasitism potential and ensure temporal precision, egg cards were replaced every 24 h for the first three days following adult emergence, as > 70% of total parasitism occurred within the initial 72 h of the adult lifespan^35^. The parasitized cards from both stored and control groups were then incubated in new jars until parasitoid emergence. Each treatment and the control were replicated 10 times.

### Effect of stored eggs on parasitoid reproductive quality (F_0_ and F_1_)

The reproductive quality of the parental generation (F_0_) reared on cold-stored eggs at different treatments was compared to the control group based on the following biological parameters:

- Parasitism rate = (Number of blackened host eggs/Total number of exposed *S. cerealella* eggs) ×100.
- Developmental period= Average duration from host egg exposure to parasitoid till adult emergence.
- Adult emergence rate = (Number of parasitized eggs with exit holes/Total number of parasitized eggs) ×100.
- Female ratio = (Number of emerged females/Total emerged adults) ×100.

Female parasitoid was identified by the antennal morphology as described by Mohamed et al. (2023)^8^.

To determine if cold storage of *S. cerealella* eggs influenced the subsequent generation (trans-generational effects), the fitness of the progeny (F_1_) was evaluated in terms of parasitism %, adult emergence %, and female proportion, under the aforementioned rearing conditions of parents. The emerged parasitoids were provided exclusively with fresh eggs (< 24 h) to prevent any carryover effects from the original cold-stored host. Each sample consisted of a piece of (2 × 1 cm) card of fresh eggs was exposed to the produced parasitoids from both stored and control groups for 24 h. This experiment was replicated 10 times per treatment.

### Statistical analysis

Data from both parental (F_0_) and offspring (F_1_) generations were analyzed using a two-way analysis of variance (ANOVA) to determine the main effects of storage temperature, duration, and their interaction on the biological parameters of *T. bactrae* parasitoid. For each specific storage temperature, a one-way ANOVA was conducted to assess the effect of storage duration. All analyses were performed within a General Linear Model (GLM) framework using R (version 4.3.1). Prior to analysis, all percentage data were subjected to arcsine square-root transformed (arcsin√x) to achieve homogeneity of variance (homoscedasticity). When significant differences were detected, mean separation was performed using Tukey’s HSD test at *p* < 0.05. Calculations were facilitated using Microsoft Excel^®^ (Analysis ToolPak). The experimental design followed a 4 × 13 factorial arrangement. Freshly laid eggs (0-day storage) were included as a control group to provide a baseline for comparison across all storage treatments. In instances where biological material was lost due to egg’s alteration or the parasitoid unable to complete development or parasitize during extended storage period or unsuitable temperature, these were treated as missing observations, and the degrees of freedom were adjusted accordingly in the Type III Sum of Squares model.

## Data Availability

All summarized data (Means ± SE) generated or analyzed during this study are included in this manuscript.
